# Tobacco industry interference to undermine the development and implementation of graphic health warnings in Bangladesh

**DOI:** 10.1136/tc-2022-057538

**Published:** 2023-04-25

**Authors:** Md Hasan Shahriar, Md Mehedi Hasan, Md Shahedul Alam, Britta K Matthes, Anna B Gilmore, A B M Zubair

**Affiliations:** 1 PROGGA: Knowledge for Progress, Dhaka, Bangladesh; 2 Department for Health, University of Bath, Bath, UK

**Keywords:** Low/Middle income country, Packaging and Labelling, Advocacy, Tobacco industry, Public policy

## Abstract

**Background:**

In Bangladesh, the 2013 Amendment of the Tobacco Control Act made graphic health warnings (GHWs) on the *upper* 50% of all tobacco packs obligatory. However, at the time of writing (May 2022), GHWs are still being printed on the *lower* 50% of packs. This paper seeks to explore how the tobacco industry undermined the development and implementation of GHWs in Bangladesh, a country known for a high level of tobacco industry interference (TII) that has rarely been examined in the peer-reviewed literature.

**Methods:**

Analysis of print and electronic media articles and documents.

**Results:**

Cigarette companies actively opposed GHWs, while bidi companies did not. The primary strategy used to influence the formulation and delay the implementation of GHWs was direct lobbying by the Bangladesh Cigarette Manufacturers’ Association and British American Tobacco Bangladesh. Their arguments stressed the economic benefits of tobacco to Bangladesh and sought to create confusion about the impact of GHWs, for example, claiming that GHWs would obscure tax banderols, thus threatening revenue collection. They also claimed technical barriers to implementation—that new machinery would be needed—leading to delays. Tensions between government bodies were identified, one of which (National Board of Revenue)—seemingly close to cigarette companies and representing their arguments—sought to influence others to adopt industry-preferred positions. Finally, although tobacco control advocates were partially successful in counteracting TII, one self-proclaimed tobacco control group, whose nature remains unclear, threatened the otherwise united approach.

**Conclusions:**

The strategies cigarette companies used closely resemble key techniques from the well-evidenced tobacco industry playbook. The study underlines the importance of continuing monitoring and investigations into industry conduct and suspicious actors. Prioritising the implementation of WHO Framework Convention on Tobacco Control Article 5.3 is crucial for advancing tobacco control, particularly in places like Bangladesh, where close government–industry links exist.

**WHAT IS ALREADY KNOWN ON THIS TOPIC**

- Graphic health warnings (GHWs) raise awareness about the dangers of smoking; they can discourage people who do not smoke from starting to smoke and increase quit intentions of those who smoke.
- In Bangladesh, GHWs were part of the 2013 Amendment of the 2005 Smoking and Tobacco Products Usage (Control) Act but remain only partially implemented as of May 2022 (time of writing).
- Several case studies from around the world document how the tobacco industry has worked against GHWs, but no study has explored tobacco industry interference (TII) in GHW development and implementation in Bangladesh, a country with a high level of TII.

**WHAT THIS STUDY ADDS**

- The paper identifies a range of strategies cigarette companies used to delay and weaken GHW guidelines and delay their implementation in Bangladesh, including working through an industry association, lobbying policymakers and public bodies, creating confusion, and taking legal action.
- Companies argued for GHWs to be printed on the lower, instead of the upper, half of tobacco packs, which was against the 2013 Amendment. They also made claims about technical barriers to delay the GHW implementation.
- Advocates engaged in several activities to push for strong guidelines and support GHW implementation.
- Although advocates were partially successful in counteracting TII, one self-proclaimed tobacco control organisation, whose nature remains unclear, sought to undermine tobacco control efforts.

**HOW THIS STUDY MIGHT AFFECT RESEARCH, PRACTICE OR POLICY**

- The study highlights the importance of continued monitoring and investigative work to better understand links between industry and government actors, as well as the nature of the organisation that created confusion in the tobacco control community.
- Implementing WHO FCTC Article 5.3 needs to become a priority to counteract TII and protect public health policymaking in Bangladesh.

## Introduction

 With over 160 million inhabitants, Bangladesh is the eighth largest cigarette market in the world.^1 2^ In 2017, around 35.3% of adults (male 46%, female 25.2%) used smoked and/or smokeless tobacco.^3^ While around one in five adults (male 16.2%, female 24.8%) consume smokeless tobacco, around 18% of adults smoke (male 36.2%, female 0.8%).^3^ Approximately three-quarters of people who smoke consume cigarettes and the rest use bidis (cheap hand-rolled cigarettes).^3^ In recent years, tobacco use has declined (43.3% in 2009, 35.3% in 2017), but remains high, and cigarette smoking rates have barely changed (14.2% in 2009, 14.0% in 2017).^4 5^ This indicates that tobacco use remains a key public health challenge in Bangladesh; every year, around 57 000 people die and around 400 000 people become disabled because of tobacco.^6^

One measure found to be effective in reducing smoking prevalence is graphic health warnings (GHWs), which warn (potential) consumers of the health risks and influence the attitudes and beliefs of those who smoke.^7^,^12^ Article 11 of the 2003 World Health Organization Framework Convention on Tobacco Control (WHO FCTC) recommends that parties enforce large, rotating GHWs on all tobacco packs.

Bangladesh was the first signee and an early ratifier of the WHO FCTC, which entered into force in 2005.^13^ In the same year, the Bangladesh government adopted the Smoking and Tobacco Products (Usage) Control Act,^14^ which included, among other measures, a provision on textual health warnings on smoked tobacco products. This was inconsistent with WHO FCTC Article 11 in terms of the type and scope of warning—the warnings were not graphical and did not cover all products. The 2005 Act also had other gaps: while it banned advertisement of tobacco products, it did not, for example, cover sponsorship in a comprehensive way. These shortcomings of the 2005 Act were addressed in a 2013 Amendment,^15^ which, among other reforms, made GHWs obligatory on the *upper* 50% of both sides of all tobacco packs and broadened the definition of tobacco products to include smokeless tobacco products. The Amendment also banned the depiction of tobacco use on television; the use of tobacco companies’ or products’ names, signs, trademarks or other symbols for sponsorship purposes; and increased fines for violating smoke-free and advertising, promotion and sponsorship provisions. To support the implementation of the Amendment, the government issued the Smoking and Usage of Tobacco Products (Control) Rules (referred to as implementation guidelines) in 2015.^16^

**Figure 1:**
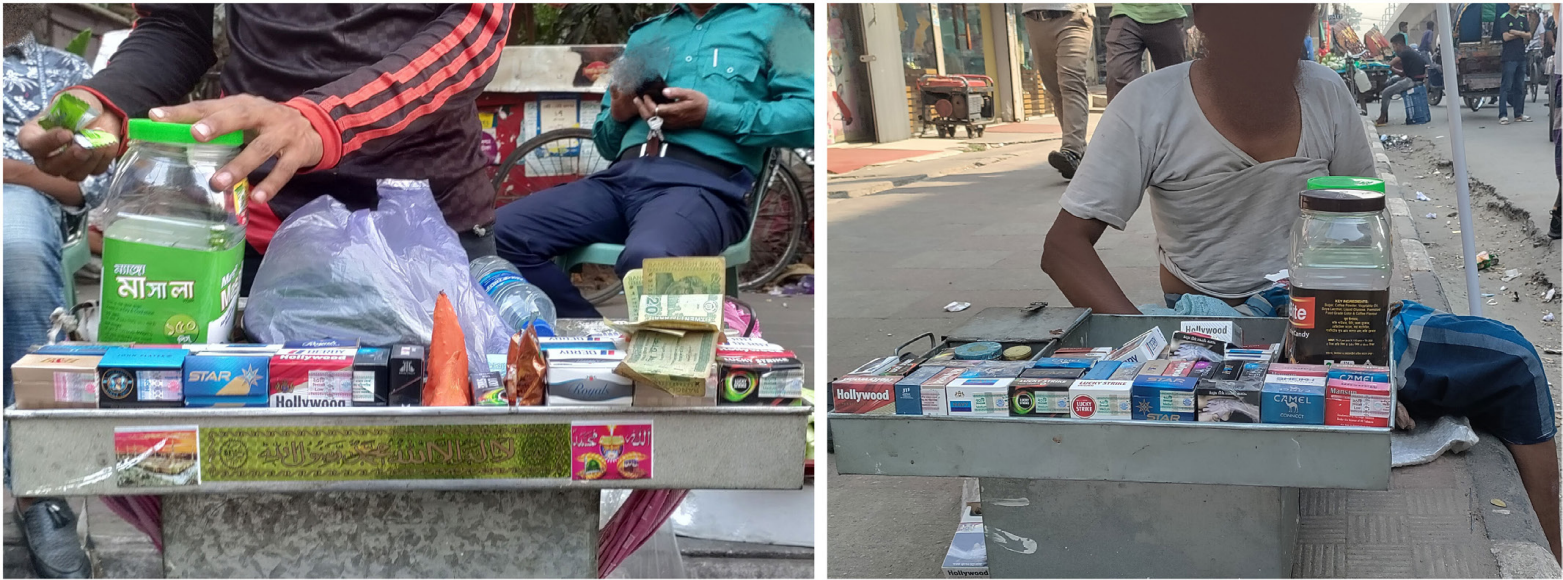
Mobile sellers offering cigarette packs in steel trays. Source: PROGGA.^53^

Despite the government’s apparent commitment to tobacco control—in 2016, the prime minister declared the country would be tobacco-free by 2040^17 18^—the application of the new policy has proven challenging, particularly regarding GHWs. The 2015 implementation guidelines gave companies six months to comply with the new requirement, but it has yet to be achieved. At the time of writing (May 2022), GHWs are still printed on the *lower*, instead of the *upper*, 50% of all tobacco packs. This placement can significantly weaken the effect of GHWs, which for someone who smokes a pack of cigarettes a day can deliver antismoking messages 7000 times per year.^8^ This is because the *lower* part of the pack is often hidden by the hand holding the pack or when the packs are being sold. In Bangladesh, tobacco products are often sold by mobile sellers who carry goods in steel trays (see figure 1), which obscure the *lower* 50% of the packs.

To date, no peer-reviewed work has focused on the development and implementation of GHWs in Bangladesh, where recent studies illustrate how close links between the tobacco industry and some government sectors hinder tobacco control progress.^19 20^ For example, in 2018, a former independent director of British American Tobacco Bangladesh (BATB), who was a high-level government official from the Ministry of Industries, became the new chairman of the National Board of Revenue (NBR),^21^,^24^ a body which repeatedly gave awards to the BATB for being a top taxpayer in the country.^22 25^ The government also holds an almost 10% share in BATB (see Box 1) and some government officials sit on BATB’s Board of Directors.^26^ The 2018–2021 Tobacco Industry Interference Indices suggest that Bangladesh faces one of the highest levels of tobacco industry interference (TII) in the South Asian region.^26^,^29^ With this study, we aim to enhance our understanding of the incomplete GHW implementation and use this as a case study to understand TII in Bangladesh. In doing so, we seek to examine which industry actors were involved; what were their targets of influence, tactics and arguments; as well as the role of tobacco control advocates in GHW development and implementation.

Box 1 **Background: the tobacco industry in Bangladesh**

- There are 12 cigarette and 117 bidi manufacturers in Bangladesh,^6^ which in financial year 2016–2017 produced an estimated 128 billion sticks of cigarettes and bidis.^5^ While the bidi market is very diverse, three large producers dominate around 97% of the cigarette market: British American Tobacco Bangladesh (BATB), the Dhaka Tobacco Company (previously owned by the local Akij Group and acquired by Japan Tobacco in 2018) and the Abul Khair Tobacco Company.^5^ BATB holds around 70% of the market.^5^ In 2016–2017, at least 8.8% of all taxes collected by the National Board of Revenue (NBR) stemmed from tobacco trade.^5^ In addition, the Bangladesh government holds an almost 10% share in BATB, distributed under different government-owned enterprises (0.33% under Bangladesh Development Bank, 6.11% under Investment Corporation of Bangladesh and 2.82% under Sadharan Bima Corporation), as well as 0.64% under the position of the President of the People’s Republic of Bangladesh.^26^,^28^

## Methods

### Data sources and collection

#### Media data

This study draws primarily on media articles, an important data source for researchers across disciplines,^30 31^ including tobacco control.^32^,^34^ Media articles are useful when conducting research on sensitive and contested issues like TII and when public access to government data is limited. The Government of Bangladesh is yet to implement WHO FCTC Article 5.3 guidelines, which require disclosure of interactions with the tobacco industry and registration of tobacco industry-affiliated entities, including lobbyists.^35^

Media articles were obtained through a pre-existing system of media monitoring established by PROGGA (Knowledge for Progress), a national think tank in Bangladesh. PROGGA has a dedicated Media Monitoring Cell that has monitored media coverage of tobacco-related issues in 59 daily newspapers, 31 internet-based media outlets, 30 broadcast media channels and two news agencies since 2011. The Media Monitoring Cell also monitors online versions of the 12 most circulated daily newspapers at the division level. For the regular monitoring, a list of keywords (e.g, tobacco control law, health warnings) in both English and Bengali is used to identify tobacco-relevant articles from these sources (see online supplemental table 1), which are then systematically categorised and stored in a database in Statistical Package for the Social Sciences (SPSS) V.23 (see online supplemental table 2).

For the present study, we focused on a period of around 4.5 years: from 1 March 2013 to 31 November 2017. This timeframe covers the two years before the 2015 implementation guidelines^16^ were gazetted, to capture their development, and around 2.5 years afterwards, including the official six-month transition period, to cover the implementation process. We extracted articles from the media monitoring database that were published during the period of interest. Potentially relevant articles were identified on the basis of the issues they covered as recorded in the database (packaging and labelling, industry influence, accountability and corporate social responsibility, tobacco law amendment), where they had been published (print or electronic media) and the type of article (news reports). In total, 5287 articles met these criteria (see online supplemental table 3).

MMH, MSA and MHS read all articles to identify those relevant to the development and implementation of GHWs. The authors regularly met to discuss the selection process, and decisions were made by consensus. In total, 161 articles were included in the study.

#### Additional documents

We obtained 11 industry, government and court documents through journalists and personal networks. These documents include correspondence from industry actors, government units and others that took place during the period of interest (March 2013–November 2017) (see online supplemental table 4). Reports published by PROGGA during the study period were also considered.

### Data analysis

**Figure 2:**
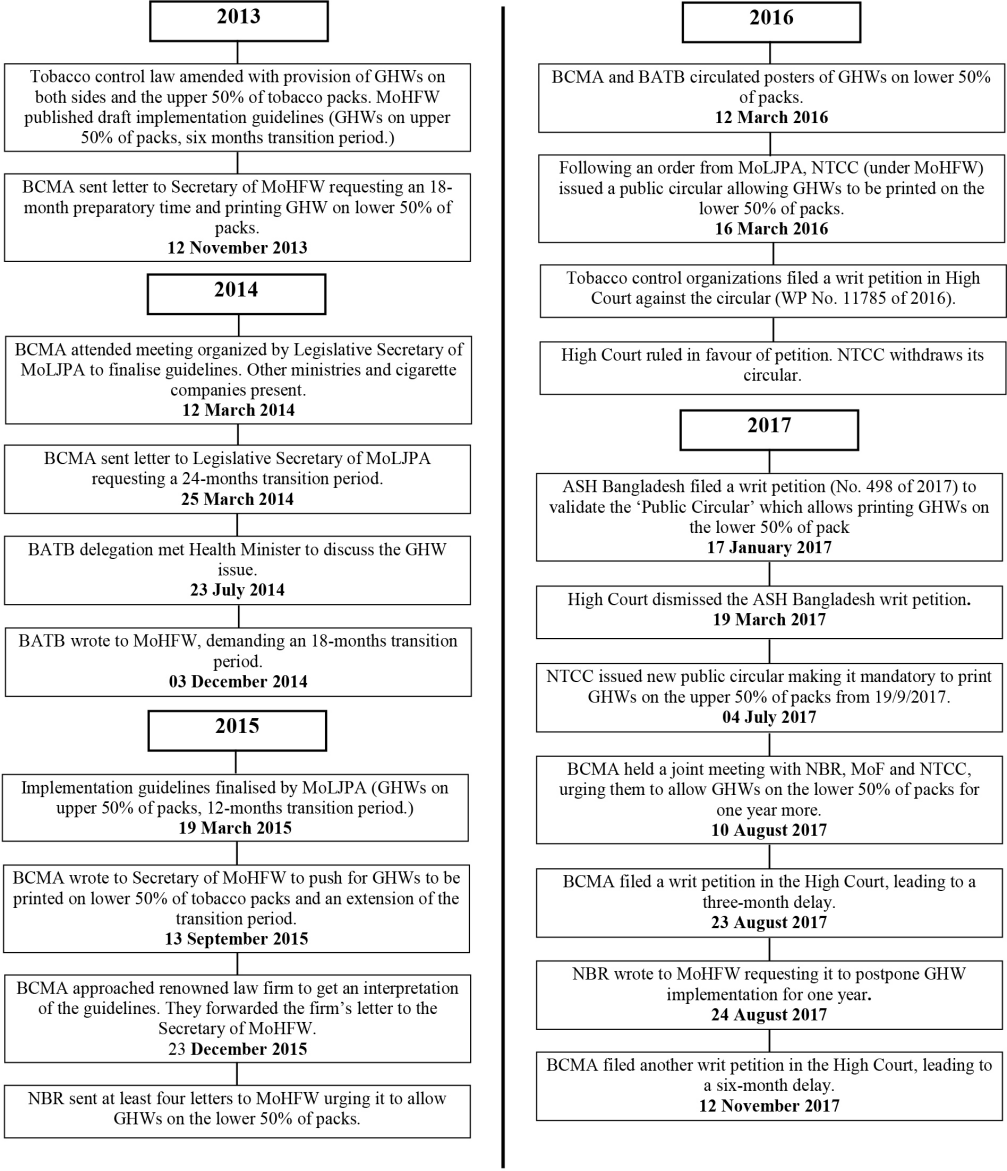
Overview of industry interference in relation to GHWs (2013–2017). ASH Bangladesh - Action on Smoking and Health Bangladesh; BATB, British American Tobacco Bangladesh; BCMA, Bangladesh Cigarette Manufacturers’ Association; GHWs, graphic health warnings; MoF, Ministry of Finance; MoHFW, Ministry of Health and Family Welfare; MoLJPA, Ministry of Law, Justice and Parliamentary Affairs; NBR, National Board of Revenue; NTCC, National Tobacco Control Cell.

Media articles and documents were read in detail and triangulated. We identified actors involved in the formulation and implementation of GHW guidelines and established a timeline (figure 2). We analysed how the tobacco industry sought to influence the process and what role tobacco control advocates played. The authors regularly met to discuss findings.

## Results

Figure 2 provides a timeline of key events in the development and implementation of GHWs, including relevant actors.

### Industry actors involved in the process

All three major cigarette manufacturers—BATB, United Dhaka Tobacco Company and Abul Khair Tobacco Company—sought to influence the process. No evidence of bidi manufacturers’ interference was found. The cigarette companies operated primarily through the Bangladesh Cigarette Manufacturers’ Association (BCMA), an organisation formed to represent and protect the interests of cigarette manufacturers. Among the companies, BATB was most active and was also the only company acting alone. As explained in more detail in the following section, the BCMA enjoyed high-level access to and maintained close communication with key government agencies, such as the Ministry of Law, Justice and Parliamentary Affairs (MoLJPA) and the Ministry of Finance (MoF), as well as the NBR. We did not find evidence of covert approaches to the government via front groups or third parties.

### Targets of influence: the government offices involved

Key ministries in the development and implementation of GHWs were the Ministry of Health and Family Welfare (MoHFW) and the MoLJPA. The former was the lead ministry for the legislation and the latter was responsible for vetting new legislation to ensure its consistency with existing laws and regulations.

Despite having no official role in GHW policymaking, the NBR became heavily involved in the process after being approached by the cigarette companies. When the gazette was published, tobacco companies raised concerns that GHWs would hamper revenue collection, a claim which facilitated the NBR’s involvement.^36 37^

### The development of the implementation guidelines

On 30 October 2013, the MoHFW published the draft implementation guidelines for the 2013 Amendment of the Smoking and Tobacco Product Usage (Control) Act on its website for public comment.^38^ The draft rules gave tobacco companies six months to comply with the Amendment, which included printing GHWs on the *upper* 50% of both sides of all tobacco packs.^38^

Shortly afterwards, the BCMA met with the secretary of the MoHFW and expressed concerns about the guidelines, which were also shared in a follow-up letter.^39 40^ The BCMA claimed that manufacturers would not be able to comply with the rules within 6 months as they needed to import machinery from Germany and France; therefore, the transition period should last 18 months.^39 40^ Furthermore, they stated that printing GHWs on the *upper* 50% of packs would be in conflict with a 2006 tax stamp and banderoles-related Statutory Regulatory Order; therefore, GHWs should be placed on the *lower* 50% of packs.^40^

In March 2014, the MoLJPA’s legislative secretary arranged a meeting of different ministries, including the MoHFW, as well as the cigarette companies, to finalise the guidelines.^41^ On invitation, the BCMA shared in writing its reasons for demanding now a 24-month transition period, explaining that due to technical limitations it would be impossible to print ‘six concurrent designs’ as the draft guidelines required.^42^ It asked for ‘realistic and implementable’ rules that considered companies’ technical capability.^42^

The MoLJPA revised the draft guidelines seemingly in response to the BCMA’s demand and extended the transition period from 6 to 18 months.^43^ This led to a tug-of-war between the MoLJPA and the MoHFW: when the MoLJPA shared the new draft with the MoHFW, the latter sent the original draft implementation guidelines back to the MoLJPA.^43^

In July 2014, a BATB delegation met the health minister, seeking to push again for a delay in the implementation of GHWs,^44^ which did not result in an amendment of the draft guidelines. The BATB also wrote another letter to the MoHFW’s additional secretary, where it raised ‘critical concerns’ regarding GHW implementation and again demanded an 18-month transition period.^45^

In January 2015, the MoHFW organised a meeting with the cigarette companies.^41^ Two months later, seemingly under pressure from the MoLJPA and the cigarette companies, the MoHFW revised the draft implementation guidelines,^46^ recommending ten (instead of six) months for the transition period. The MoLJPA added 2 more months, setting the transition period at 12 months.^47^ On 19 March 2015, around 1.5 years after the draft was first publicised, the guidelines were gazetted, requiring GHW implementation by 19 March 2016.^48^

### Industry efforts to prolong the transition period and weaken GHWs

Around halfway through the transition period, the cigarette companies sought to extend the deadline once more: in September 2015, the BCMA sent a letter to the MoHFW claiming that it had only learnt about the finalisation of the GHW image selection through a financial newspaper (*The Financial Express*) and had not received an electronic copy of the images.^49^ Its members should be allowed a 12-month transition period following receipt of the images.^49^ The letter also repeated its demand for GHWs to be printed on the *lower* 50% of packs, claiming this was necessary for compliance with the VAT (Value Added Tax) Act 1991, which requires banderols (strips of machine-readable tax stamps) vertically affixed around the *upper* part of cigarette packs.^50^ The 2013 Amendment did not specify the placement of banderols; it only required them not to cover GHWs.^38^ The BCMA also reiterated that more time was needed to prepare for implementation, including for importing new printers and training staff.^50^ Raising the same issues, the BCMA wrote another letter to the MoHFW’s secretary.^51^ It also claimed that GHWs, by obscuring banderols, could affect national revenue collection.

The BCMA also approached a renowned law firm for an interpretation of the implementation guidelines and forwarded the letter to the secretary of the MoHFW.^52^ The letter claimed, for example, that printing GHWs on the *lower* 50% of packs would still comply with the 2013 Amendment.^52^ Furthermore, it argued that if GHWs were printed on the *upper* 50% of packs, these would be hidden by banderols and tax stamps, which would be in conflict with the implementation guidelines.^52^

The NBR also put pressure on the MoHFW to allow GHWs on the *lower* 50% of packs. It sent at least four letters regarding the issue and organised a meeting with cigarette company representatives and the MoHFW in late 2015.^36^ Echoing the BCMA’s claims, it also repeatedly stressed the ‘detrimental effect’ of printing GHWs on the *upper* 50% of packs’, claiming that it might hurt government revenue collection.^36^

To address the concerns regarding the placement of banderols in relation to GHWs which the BCMA and the NBR had raised, the MoHFW recommended that tax stamps and banderols to be attached to the side of the packs.^53^ Ignoring this recommendation, the MoLJPA signed an order requiring GHWs to be printed on the *lower* 50% of packs.^54^ The National Tobacco Control Cell (NTCC), a body under the MoHFW, issued a public circular on the same day, announcing GHWs would be printed on the *lower* 50% of tobacco packs.^55^ The order came into force as scheduled on 19 March 2016, but the placement of the GHWs was in contradiction to the 2013 Amendment and the implementation guidelines adopted the previous year.

**Figure 3:**
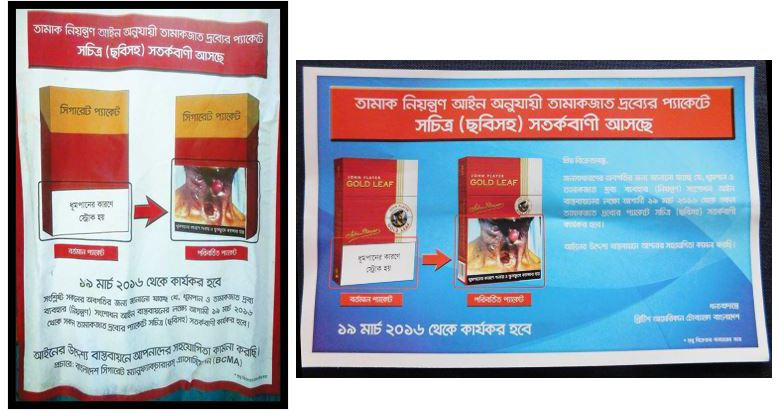
Posters circulated by the Bangladesh Cigarette Manufacturers’ Association and British American Tobacco Bangladesh on 12 March 2016. Source: PROGGA.^53^T

Before the order had been signed by the minister of the MoLJPA and published in the gazette, the BCMA and the BATB had circulated posters (see figure 3) all over the country that stated that GHWs would be printed on the *lower* 50% of packs.^53^ This suggests that they already knew of the decision before it had been made public.

### Tobacco control advocates push for GHW implementation

Tobacco control advocates closely monitored the entire process and repeatedly voiced concerns about the delay and weakening of the policy and the indsutry influence on the process.^56^ For example, Saber Hossain Chowdhury MP, then chairman of the Inter-Parliamentary Union and convenor of Tamakmukto Bangladesh Mancha (Tobacco-free Bangladesh Platform, a coalition of antitobacco organisations), had written a letter to the health minister in late 2015, where he expressed concerns about the length of the process. He suggested that rather than shifting GHWs to the *lower* 50% of packs, stamps and banderols should be attached to the sides of the packs.^57 58^ This was later proposed by the MoHFW, but disregarded by the MoLJPA (see above).

To push for a timely implementation of GHWs and expose industry attempts to weaken the rules, tobacco control advocates also engaged in nationwide public events and worked with earned media, social media campaigns and community radio stations to bring more attention to the issue.^59^ In March 2016, nine days before the end of the transition period, a day-long exhibition of the GHW images set by the MoHFW was organised in front of the Bangladesh National Museum in Dhaka, the capital. The event was attended by members of parliament and representatives from civil society. Dummy cigarette packets with GHWs were exhibited in different parts of the country.^59^ Around the same time, tobacco control groups organised a discussion event on GHWs which was chaired by the health minister, and journalists from all major mainstream media platforms participated.^59^

In addition, in the last weeks of the transition period, at least six human chain events were organised in six districts, and road shows along with mobile musical concerts took place in the capital^54^ and other divisional capitals.^59^ Furthermore, a commercial on GHW implementation was aired on all community radio stations across Bangladesh just before the transition period ended.^59^

### Further delay and confusion: civil society and cigarette companies going to the High Court

Tobacco control advocates heavily criticised the NTCC’s move to issue a public circular in line with the MoLJPA’s order, which contradicted the guidelines set by its controlling ministry (MoHFW). Some tobacco control organisations (PROGGA, UBINIG and Pratyasha) issued a writ petition (no 11785 (2016)) to the High Court questioning the legality of NTCC’s circular.^60^ In its ruling, the High Court agreed with the concerns raised, which led the NTCC to withdraw the circular.^61^

However, discord within the coalition emerged when Action on Smoking and Health (ASH) Bangladesh filed a writ petition (no 498 (2017)) to validate NTCC’s public circular, arguing—echoing the cigarette companies’ position—that GHWs should be printed on the *lower* 50% of packs.^61^ They accused the MoHFW and the NTCC of ‘illegal and arbitrary actions’. The High Court dismissed ASH Bangladesh’s petition due to the lack of ‘necessary logic and evidence’.^55^ The High Court also stated that although ASH Bangladesh had been ‘established with an aim to reducing tobacco manufacturing and consumption’, it was ‘clear’ from the petition that it ‘has come in aid of the tobacco manufacturers’.^55^ More concerns about the nature and role of ASH Bangladesh were raised: a report showed that the organisation was registered in 2003, but only became active in February 2017 in the context of the GHWs.^61^ There is no evidence that ASH Bangladesh is linked to genuine tobacco control organisations of the same name.

In line with the High Court’s stance, the NTCC issued a new public circular in July 2017, which made it mandatory to print GHWs on the *upper* 50% of packs from 19 September 2017, consistent with the 2013 Amendment and the implementation guidelines.^55 62^

The BCMA held a joint meeting with the NBR, MoF and MoHFW, urging them to allow GHWs on the *lower* 50% of packs for one additional year.^37^ As before, the NBR took an industry-friendly approach: it sent a letter to the MoHFW highlighting the cigarette companies’ importance in generating government revenue and requesting a 12-month extension to provide cigarette companies time to import the machinery needed.^37^ The BCMA also filed two writ petitions in the High Court against the implementation guidelines in August and November 2017,^63^ which led to further delay.^64 65^ At the time of writing (May 2022), GHWs are still printed on the *lower* 50% of packs.

## Discussion

This study explored the development and implementation of GHWs in Bangladesh following the 2013 Amendment of the Tobacco Control Act. It found that only cigarette manufacturers, no bidi manufacturers, sought to influence the process. The cigarette companies used several techniques to significantly delay the development and implementation of the guidelines, including working through a business association (BCMA), lobbying policymakers and government bodies (in particular the NBR), distorting information (eg, by obtaining and using a letter from a lawyer), and using legal tools such as writ petitions. These techniques are consistent with industry efforts to undermine health warnings^66^,^69^ and other tobacco control policies elsewhere.^70^,^72^ BATB was the most active company and the only one which acted alone. Deploying common industry arguments, the BCMA and BATB repeatedly claimed that the implementation guidelines would contravene existing laws and pointed to potential (macro)economic losses, arguing that revenue collection might be hampered if banderols were obscured.^70 71^ They sought to delay GHW implementation by pointing to technical barriers, including the lack of equipment, which tobacco companies had claimed before, particularly in Low and Middle Income Country (LMICs).^71^ Although the BCMA has repeatedly highlighted the issue of illicit trade—one of the most common arguments for opposing tobacco control regulation in LMICs^71^—in discussions on tobacco taxation in Bangladesh,^73^ it was not raised directly in this case. However, it was arguably implicit in the banderols/revenue argument. The importance of arguments around revenue collection is unsurprising given what we know from other countries; however, as elsewhere,^74^ the industry’s excise duties do not cover the costs tobacco imposes on Bangladeshi society.^75^

The study also shows how industry argument-based and action-based strategies are tailored to a specific context. The insistence on the placement of GHWs on the *lower*, instead of the *upper*, part of tobacco packs may be particularly relevant where mobile selling is common. However, it may still be important elsewhere; even in shops, the *lower* part of a pack may be less visible. Furthermore, using the revenue authority (NBR) to exercise pressure was facilitated by an established link. For example, following the NBR’s invitation, the BCMA had repeatedly attended prebudget meetings and presented its proposals regarding tobacco taxation and other tobacco control measures.^73^ The relevance of this strategy goes beyond budgetary tobacco pricing, tax measures and GHWs: more recently, the NBR, apparently acting on the industry’s behalf, sought to undermine the national tobacco control policy^76 77^ and helped the industry to secure special permission to continue producing cigarettes during the COVID-19 pandemic.^78^

This study both illustrates and supports recent findings on the stalled implementation of WHO FCTC Article 5.3 in Bangladesh, documenting extensive conflicts of interests among crucial government actors and institutional constraints on those seeking to strengthen tobacco control.^20^ The government’s share in BATB and the presence of high-level officials on the company’s Board of Directors are particularly concerning.^26^,^28^ The findings also illustrate how bureaucratic processes can favour the tobacco industry: the complex procedures delay in involving various government organisations helped the cigarette companies create conflict and confusion between these agencies, significantly delaying GHW development and implementation.^79^

As in other LMICs,^80 81^ civil society organisations played key roles in exposing and countering cigarette companies’ efforts to weaken and delay GHW implementation. They combined several strategies: they used various media channels (press conferences, social media and radio) and organised public events such as road shows and human chains, as well as events that targeted policymakers more directly. However, they could not prevent the significant delay in implementation and were unsuccessful in convincing policymakers to respect WHO FCTC Article 5.3. The study further shows that discord and confusion within the tobacco control community can also undermine advocates’ efforts to push for strong tobacco control rules. A strong and united tobacco control coalition facilitates countering TII and protecting public health policies.^29 82^ The unclear nature of ASH Bangladesh makes it challenging for advocates to address the organisation’s conduct and its effects. This underlines the centrality of investigative and monitoring activities for tobacco control.^82^

This study is the first to examine TII relating to GHWs in Bangladesh, but has a number of limitations. First, it relies on media articles and a limited number of additional documents. Full documentation of the meetings between the government and the industry, for example, would have provided a fuller picture of the process. Key informant interviews could also have added to the study. Second, the 4.5-year study duration means that it does not capture GHW policymaking and implementation in its entirety. We sought to cover key moments in the process, while ensuring that the number of articles to be reviewed was manageable. Finally, some authors are from PROGGA, which has been actively involved in the process and features in the findings. Some of PROGGA’s publications are also referenced. This could introduce bias into the analysis. We sought to address this by triangulating different data sources.

## Conclusion

To our knowledge, this is the first peer-reviewed paper on TII linked to GHWs in Bangladesh. It documents how cigarette companies interfered in the development and implementation of GHWs. Over the seven years after the implementation guidelines that require GHWs to be placed on the *upper* 50% of packs, were gazetted, GHWs are still printed on the *lower* 50% of packs. Cigarette companies’ interference was facilitated by close ties with a government body that was not formally involved in GHW implementation. Tobacco control advocates were critical in countering TII but faced challenges when one self-proclaimed tobacco control organisation, whose nature remains unclear, sought to undermine their efforts. Continued monitoring of industry interference and suspicious activities is needed. Furthermore, implementing WHO FCTC Article 5.3 must become a priority to counteract industry interference and protect public health. Otherwise, tobacco control progress is likely to remain slow in Bangladesh.

## supplementary material

10.1136/tc-2022-057538 online supplemental file 1

## Data Availability

No data are available.
