# Supplementary material for: Tobacco industry interference to undermine the development and implementation of graphic health warnings in Bangladesh
Source: Tob Control. 2023 Apr 25;33(5):e057538. doi: 10.1136/tc-2022-057538 (PMC11503176; doi:10.1136/tc-2022-057538)
Supplement: online supplemental file 1 [file tobaccocontrol-33-5-s001.pdf]

Supplemental material

Table 1: List of keywords used by the Media Monitoring Cell to identify relevant media articles then stored in the media monitoring database*

Keywords used as search criteria
Tobacco
Tobacco industry
Smoking
Tobacco Industry Interferences
Tobacco Control Act
Smoking-free
Tobacco Control Law
Tobacco Control Rules BAT(B)
Graphic Health Warning
Packaging and labeling GHW
Anti-Tobacco Media Alliance
Cigarette
Bidi
Bangladesh Cigarettes Manufacturing Association/BCMA

* Bengali words with the same meaning were also scrutinised.

Table 2:

The Media Monitoring Cell systematically categorises and digitally stores tobacco-relevant coverage detailing the following:
a) Date of publication
b) ID of the report
c) Issues covered (see Table 3.1)
d) Stance of the coverage (i.e., pro-tobacco, anti-tobacco or unclear stance)
e) Type of media (print, electronic or broadcast)
f) Type of coverage (i.e., reports, event news, articles/features, opinions)
g) The week of the month in which it was published. If a report is published within first 1 to 7 days of the month, it is put under 'Week 1'. Accordingly, reports published are also marked as week-2, week-3, week-4 and week-5 (for day 29-31 of a month).

Table 3: Identification of media articles

Table 3.1: Total number of Articles Collected via Media Mentoring Cell

Issues Covered	Pre-enactment* (3/2013-3/2015)	Post-enactment* (3/2015-11/2017)	Total
Smoke-free	1681	3202	4883
TAPS	623	876	1499
Tobacco Price and Tax	2033	7944	9977
**Packaging and Labeling**	**141**	**1941**	**2082**
**Industry Influence, Accountability, CSR**	**702**	**1206**	**1908**
Tobacco and Health	2211	6245	8456
Tobacco Companies	223	274	497
**Tobacco Law Amendment**	**809**	**1064**	**1873**
Tobacco Cultivation	360	651	1011
Event/meeting Coverage	1023	1816	2839
Illicit tobacco trade	0	1621	1621
Miscellaneous	1367	347	1714
**Total**	**11173**	**27187**	**38360**

Table 3.2: Extracting Articles based on Issues, Type of Media, and Type of Media Coverage

Selected 'Issues Covered'	Pre-enactment (3/2013-3/2015)	Post-enactment (3/2015-11/2017)	Total
Packaging and Labeling	141	1941	2082
Tobacco Industry Influence, Accountability, CSR	702	1206	1908
Tobacco Law Amendment	809	1064	1873
**Total**	**1652**	**4211**	**5863**

Type of Media	Pre-enactment (3/2013-3/2015)	Post-enactment (3/2015-11/2017)	Total
**Print**	**817**	**1656**	**2473**
**Electronic (Web-based)**	**745**	**2277**	**3022**
Broadcast (TV)	90	278	368
**Total**	**1652**	**4211**	**5863**

Type of Media Coverage	Pre-enactment (3/2013-3/2015)	Post-enactment (3/2015-11/2017)	Total
**Report**	**1463**	**3824**	**5287**
Event news	1	1	2
Articles/features	47	60	107
Opinion	27	13	40
Editorial	12	12	24
Talk show	1	0	1
Pictorial	11	23	34
**Total**	**1562**	**3933**	**5495**

Reports about Specific Category	Pre-enactment (3/2013-3/2015)	Post-enactment (3/2015-11/2017)	Total
Packaging and Labeling	129	1756	1885
Tobacco Industry Influence, Accountability, CSR	640	1106	1746
Tobacco Law Amendment	694	962	1656
**Total**	**1463**	**3824**	**5287**

*The guidelines were gazetted on 19/03/2013.

Table 4: Documents

Number	Date	Author	Summary	Reference in text
**1.**	12 NOV 2013	Bangladesh Cigarette Manufacturers' Association (BCMA)	Addressed to the Health Secretary, the letter claimed that some provisions of the implementation guidelines would not be implementable and might create confusion.	40
**2.**	25 MAR 2014	Bangladesh Cigarette Manufacturers' Association (BCMA)	Addressed to the Law, Justice and Parliamentary Affairs Secretary, the letter was a "technical note highlighting key concerns on GHW implementation".	42
**3.**	21 JUL 2014	British American Tobacco Bangladesh (BATB)	Minutes of the BATB Board's CSR Committee, attended by BATB Chairman, Managing Director and Non-Executive Directors.	44
**4.**	3 DEC 2014	British American Tobacco Bangladesh (BATB)	Addressed to the Additional Secretary of Ministry of Health and Family Welfare, the letter raised "critical concerns pertaining to implementation of the Clause 9 of the proposed implementation guidelines".	45
**5.**	13 SEP 2015	Bangladesh Cigarette Manufacturers' Association (BCMA)	Addressed to the Additional Secretary of Ministry of Health and Family Welfare, the letter sought, among others, clarification regarding electronic files GHWs.	49
**6.**	20 DEC 2015	Rokanuddin Mahmud and Associates (A law firm)	Addressed to the Chairman of BCMA, the letter is a response to BCMA's request for a "legal opinion" regarding the placement of GHWs.	52
**7.**	23 DEC 2015	Bangladesh Cigarette Manufacturers' Association (BCMA)	Addressed to the Health Secretary, the letter claimed that it would be impossible for cigarette companies to print GHWs by 19 March 2016 and recommended that authorities allow companies to print GHWs on lower half of tobacco packs.	51
**8.**	10 MAR 2016	National Board of Revenue (NBR)	Addressed to the Health Secretary, the letter emphasised the revenues generated by the tobacco industry and claims that printing GHWs on upper half of pack would get in the way of attaching banderoles. It also urged the Health Ministry to allow GHWs on the lower half of packs.	36

**9.**	24 AUG 2017	National Board of Revenue (NBR)	Addressed to the Health Secretary, the letter quoted "cigarette manufacturing entities" to say that affixing banderoles properly and printing GHWs on the upper half of tobacco packs requires to import new machine from abroad. The letter requested the Health Ministry to postpone the effectiveness of its public notice by one year, "considering the importance of cigarette industry in generating government revenue and the lack of technology and necessary machinery to affix stamps and banderoles on cigarette packs".	37
**10.**	23 AUG 2017	The Supreme Court of Bangladesh (High Court Division)	In this document, the petitioner is BCMA and the respondents are the Secretary, Ministry of Finance; the Secretary, Ministry of Law, Justice and Parliamentary Affairs; the Secretary, Ministry of Health and Family Welfare; NBR represented by its Chairman, Additional Secretary, the Ministry of Health and Family Welfare; Secretary, Legislative and Parliamentary Affairs Division; Joint Secretary and Coordinator, National Tobacco Control Cell (NTCC).<br><br>It says, "let the operation of the public notice [regarding printing GHWs on upper half of packs by the Health Ministry] ... be stayed [i.e., stay order] for a period of three months from date and the respondents are directed to allow the members of the petitioner to publish the GHWs in the lower half of cigarette packet."	64
**11.**	12 NOV 2017	The Supreme Court of Bangladesh (High Court Division)	The document extended the stay order: "Let the stay order granted earlier by this Court be extended for a further period of six months from date".	65
